# Comparison of lectin binding of drusen, RPE, Bruch’s membrane, and photoreceptors

**Published:** 2009-05-04

**Authors:** Yvonne B. D’Souza, Carolyn J.P. Jones, Richard E. Bonshek

**Affiliations:** 1 Academic Unit, Manchester Royal Eye Hospital, Manchester, UK; 2 Maternal and Fetal Health Research Centre, University of Manchester, Manchester, UK; 3 National Specialist Ophthalmic Pathology Service (NSOPS), Manchester Royal Infirmary, Manchester, UK

## Abstract

**Purpose:**

Drusen are deposits located between the retinal pigment epithelium and Bruch’s membrane in age-related maculopathy. They are believed to be photoreceptor byproducts that are incompletely metabolized by the retinal pigment epithelium. This study therefore compares the lectin histochemistry of drusen, photoreceptors, retinal pigment epithelium, and Bruch’s membrane.

**Methods:**

Semithin sections of three eyes with age-related maculopathy were studied using 19 biotinylated lectins and an avidin-peroxidase-revealing system with and without neuraminidase pretreatment.

**Results:**

High mannose, bi and tri-antennary nonbisected and bisected complex N-glycan, N-acetyl glucosamine and galactose were expressed by drusen, retinal pigment epithelium, Bruch’s membrane, and photoreceptors while N-acetyl galactosamine and fucose were absent; treatment with neuraminidase exposed subterminal galactose in both sites and sparse N-acetyl galactosamine residues in drusen alone. Overall, there were striking similarities between the lectin binding of drusen, retinal pigment epithelium, and the photoreceptor outer segments, though cone outer segments were distinct in some features of their O-linked glycosylation.

**Conclusions:**

The results suggest that the pathogenesis of drusen is a combined mechanism, involving photoreceptors, Bruch’s membrane, and the retinal pigment epithelium.

## Introduction

Drusen are yellowish extracellular deposits between the retinal pigment epithelium (RPE) and the inner collagenous zone of Bruch’s membrane in the aging eye [1,2]. Ultrastructurally, the material in early drusen resembles components of the aging Bruch’s membrane, both containing membrane-bound bodies that rupture to release vesicular and granular material [3]. Previous studies have suggested the role of photoreceptors and RPE in the formation of drusen [4].

Bishop and coworkers [5] have detected complex bisected and non-bisected bi and tri-antennary *N*-glycans in the human interphotoreceptor matrix and photoreceptor inner and outer segments as well as sialic acid in 2,3 and to a lesser extent 2,6 linkage with subterminal N-acetyl galactosamine (GaINAc) in interphotoreceptor matrix. In their study, rod and cone outer segments bound mannose while rod outer segments bound N-acetyl glucosamine (GlcNAc). Fong and colleagues [6] have previously described interphotoreceptor retinol binding protein binding to *Canavalia ensiformis* (CON-A), *Lens culinaris* (LCA), and wheatgerm (WGA) agglutinins and after pretreatment with neuraminidase and *Ricinus communis* agglutinin (RCA). The cone matrix sheath binds the lectin *Arachis hypogaea* (AHA) while the rod matrix binds WGA, LCA, and AHA after neuraminidase pretreatment. Uehara and colleagues [7] have identified sialylated and nonsialylated Jacalin binding protein in association with rod and cone cells respectively. They postulate that this protein is synthesized and acts locally. The hypothesis of this study is that drusen pathogenesis is a combined mechanism, involving photoreceptors, Bruch’s membrane, and RPE. We have therefore compared the saccharide composition of Bruch’s membrane, RPE, and photoreceptors with that of drusen using lectins as a tool. Lectins are glycoproteins with a high affinity for specific saccharides. As the lectin binding specificities are known, the presence of particular saccharides can be inferred. Semithin, resin-embedded tissue sections were used to enhance resolution at the light microscope level

## Methods

Three paraffin-embedded eyeball specimens were obtained from the pathology archives of the Manchester Royal Eye Hospital for this study. The histological diagnosis in all three cases was age-related macular degeneration. The clinical diagnosis for two of these cases, and the reason for enucleation, was blind painful eyes due to coexistent glaucoma. The third case was a blind eye in which the patient developed a corneal abscess. In all cases, drusen were an associated finding on histopathological examination.

Small pieces (5x3 mm) of tissue were excised from wax blocks. These were dewaxed in vials of xylene then rehydrated and washed in 0.1 M sodium cacodylate buffer, pH 7.3, before fixation in 2.5% glutaraldehyde (Agar Scientific Ltd., Stansted, UK) in 0.1 M sodium cacodylate buffer, pH 7.3, for 2–4 h. Afterwards, samples were rinsed in buffer and processed in TAAB epoxy resin (TAAB Laboratories Equipment Ltd., Aldermaston, UK) the next day.

Suitable areas containing drusen from blocks of all 3 specimens were identified on light microscopy using 0.5 µm thick sections stained with 1% (w/v) toluidine blue in 1% (w/v) aqueous sodium tetraborate. Following identification of appropriate areas, further sections 0.75 µm in thickness were cut and mounted on 3′aminopropyltriethoxysilane-coated slides, dried at 50 °C for 48 h and stained with lectins as previously described [8]. Resin was removed with saturated sodium ethoxide diluted 1:1 with absolute ethanol for 15 min and, after washing in ethanol followed by distilled water; endogenous peroxidase was blocked with 10% (v/v) hydrogen peroxide (100 volumes, BDH, Poole, UK) before exposure to 0.03% (w/v) trypsin (Type II-S, Sigma, Poole, UK) in 0.05 M Tris buffered saline (TBS), pH 7.6, for 4 min at 37 °C. The trypsin was used at 37 °C for 4 min. For relevant lectins (SNA-1, MAA. LFA, WGA, AHA, ECA, Jacalin, and SBA) the terminal sialic acid was removed by pretreatment with neuraminidase (Sigma, type VI from *Clostridium perfringens*), 0.1units/ml in 0.2 M sodium acetate buffer, pH 5.5 containing 1% calcium chloride for 1 h at 37 °C to expose the subterminal sugar [9]. After washing in distilled water followed by a 0.05 M TBS wash, sections were incubated with 10 µg/ml biotinylated lectin in 0.05 M TBS containing 1 mM calcium chloride for 1 h at 37 °C. A panel of 19 biotinylated lectins (Table 1 and references [10-12]) known to bind saccharides present in mammals was used. After washing in TBS with calcium chloride buffer, the sections were treated with 5 µg/ml avidin peroxidase (Sigma) in 0.125 M TBS, pH 7.6, with 0.347 M sodium chloride for 1 h at 37 °C [13]. Sections were washed and sites of lectin binding revealed with 0.05% (w/v) diaminobenzidine tetrahydrochloride dihydrate (Aldrich Chemical Co., Gillingham, UK) in 0.05 M TBS, pH 7.6, and 0.015% (v/v) hydrogen peroxide (100 volumes) for 5 min at 18 °C±0.5 °C. Sections were rinsed, air-dried, and mounted in neutral synthetic mounting medium (BDH) without counterstaining.

**Table 1:** Biotinylated lectins used and their major specificites

**Lectin**	**Source**	**Major specificity**
**Mannose**		
CON A	Canavalia ensiformis	aD-man,a-D-Glc, terminal or a1,2 N-linked sequences, high mannose, intermediate and small complex structures [10].
PSA	Pisum sativum	a-D-man in non-bisected bi and tri antennary complex N glycan [10].
e-PHA	Phaseolus vulgaris (erythroagglutin)	Bisected bi and tri-antennate complex N glycan [10]
l-PHA	Phaseolus vulgaris (leukoagglutinin)	Tri and tetra-antennate nonbisected complex N glycan [10].
**Sialic acid**		
SNA-1	Sambucus nigra	Neu5Aca2,6Gal/GalNAc- [10].
MAA	Maackia amurensis	Neu5Aca2,3Galb1 [10]
LFA	Limax flavus	Sialyl terminals [11]
**N-acetyl glucosamine oligomers**		
WGA	Triticum vulgaris	(−4GlcNAc b1,4GlcNAcb1-)n,
		(-Galb1,4GlcNAcb1-)n [10]
DSA	Datura stramonium	-GlcNAcb1,4GlcNAc- [10]
STA	Solanum tuberosum	b1,4GlcNAc oligomers [10]
		N-Acetyl galactosamine
SBA	Glycine max	Terminal GalNAca1– [10]
DBA	Dolichos biflorus	GalNAca1,3(Fuc1,2)Galb1,4GlcNAc- [10]
WFA	Wisteria floribunda	GalNAcα1,6Galβ1-, bGalNAc [12]
**Galactose**		
AHA	Arachis hypogaea	Galb1,3GalNAca->Galb1,4GlcNAca1 [10]
MPA	Maclura pomifera	Galb1,3GalNAca1->GalNAca1– [10]
Jacalin	Artocarpus integrifolia (Jacalin)	Galb1,3GalNAc-, Gala1,6– [10]
BSA-1B4	Bandeiraea simplicifolia	Gala1,3Galb1,4GlcNAcb- [10]
ECA	Erythrina cristagalli	Galb1,4GlcNAc- [10]
**Fucose**		
UEA-1	Ulex europaeus	Fuca1,2Galb1,4GlcNAc- [10]

The biotinylated lectins used in the study are listed grouped by major specificity; source references are provided in parentheses.

Kidney tissue from a bank of normal control tissues held in the archives of the Histopathology Department, Manchester Royal Infirmary was used as a positive control while negative controls were obtained by substitution of the lectin with buffer alone. Confirmation that the lectins were binding via their binding sites was obtained by incubating the lectin in the presence of 0.2 M of the appropriate competing sugar, while reduction or abolition of staining by sialic acid-binding lectins following neuraminidase predigestion was taken as confirmation of lectin specificity for sialyl residues. Sections were examined using an Olympus BX51 (Olympus UK Ltd, Watford, UK) light microscope by two coauthors (YD and REB), and a semiquantitative ranking system was used to score the intensity of staining: no staining (0), weak (1), moderate (2), strong (3), and intense (4).

## Results

**Figure 1:**
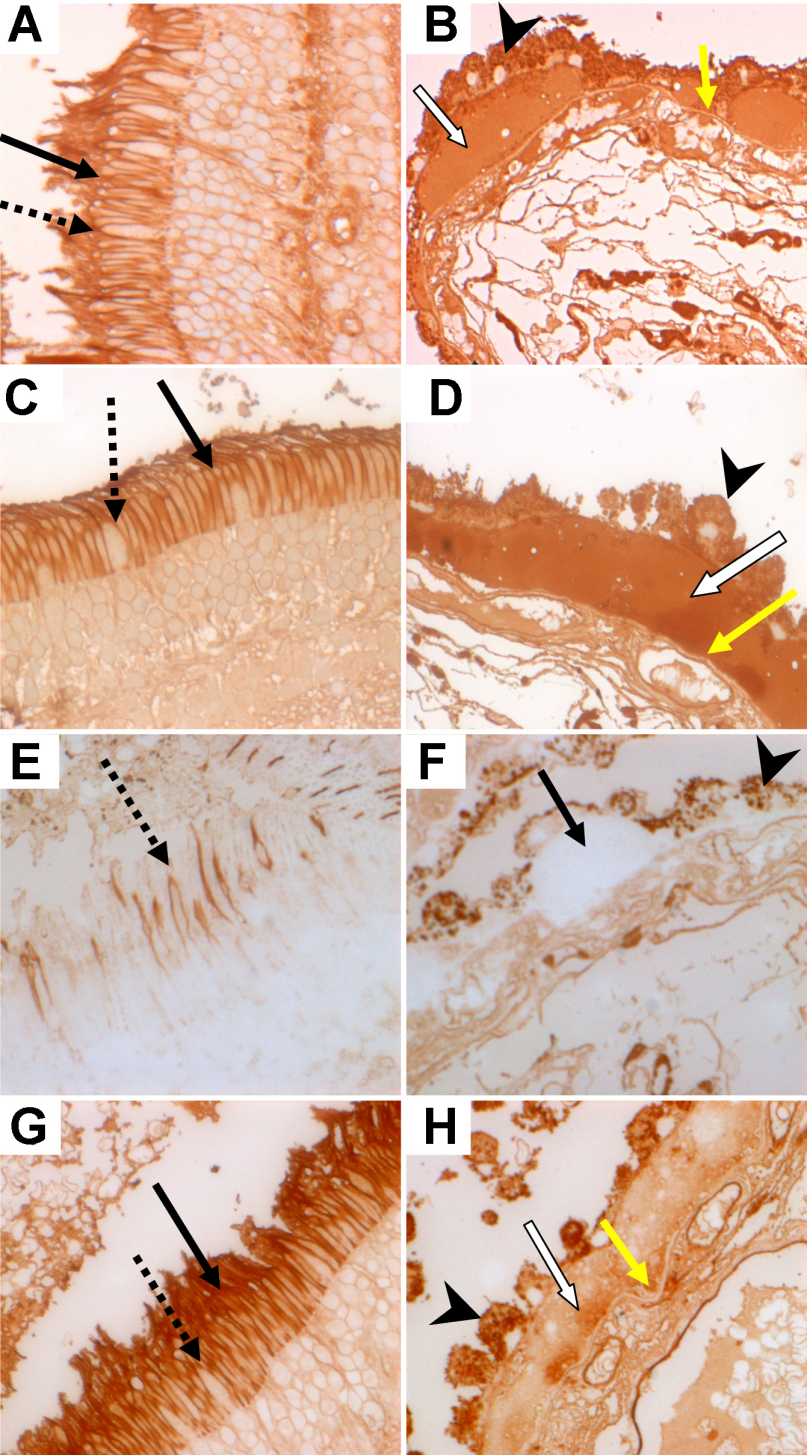
Comparison of the lectin-binding pattern of drusen, RPE, Bruch’s membrane, and photoreceptors. **A**: PSA binds outer segments and cell membranes of both cones (dotted arrow) and rods (solid arrow) strongly. **B:** PSA binds drusen (white arrow) and Bruch’s membrane (yellow arrow) strongly. **C:** MPA binds outer segments and cell membranes of both cones (dotted arrow) and rods (solid arrow) with moderate intensity. **D:** MPA binds drusen (white arrow) and Bruch’s membrane (yellow arrow) with moderate intensity. **E:** AHA without pretreatment with neuraminidase binds outer segments and cell membranes of cones (dotted arrow) but not rods. **F:** AHA without pretreatment with neuraminidase does not bind drusen (arrow) or Bruch’s membrane. **G:** After pretreatment with neuraminidase, AHA binds outer segments and cell membranes of both cones (dotted arrow) and rods (solid, black arrow) strongly and (**H**) drusen (white arrow) with moderate intensity. Arrowhead indicates RPE. Original magnification is 600×

Eleven lectin probes (CON A; PSA; (Figures 1A,B); ePHA; WGA, DSA, STA, MPA (Figures 1C,D), Jac, LFA, SNA-1, and MAA) bound drusen, RPE, Bruch’s membrane and photoreceptors. All the abbreviations used are standard nomenclature for lectins and are listed in Table 1. lPHA, DBA, UEA-1, BSA-1B_4_, ECA, and SBA did not bind any of the structures. AHA (Figures 1E,F) and WFA bound cone outer segments but not drusen (Table 2). Similar binding patterns were noted in hard, soft, and confluent drusen. It was difficult to comment on the interphotoreceptor matrix with accuracy due to lack of resolution at the light microscopic level.

**Table 2:** Comparison of lectin binding of drusen, RPE, Bruch’s membrane, and photoreceptors in AMD

**Lectin**	**Drusen**	**RPE**	**Bruch’s**	**RC**	**CC**	**Rod OS**	**Cone OS**	**RM**	**CM**
CON A	3	3	3	2	0	3	3	3	3
PSA	3	3	3	1	0	3	3	3	3
ePHA	3	3	3	1	0	3	3	3	3
lPHA	0	0	0	0	0	0	0	0	0
SNA-1	4 (4)	4 (4)	4 (4)	1 (0)	0 (0)	4 (0)	4 (0)	0 (0)	0 (0)
MAA	3 (1)	3 (0)	0 (0)	1 (0)	0 (0)	3 (3)	3 (3)	3 (3)	3 (3)
LFA	3 (0)	1 (0)	0 (0)	1 (0)	1 (0)	3 (0)	3 (0)	1 (0)	1 (0)
WGA	2 (2)	2 (0)	2 (0)	0 (0)	0 (0)	2 (0)	2 (0)	2 (0)	2 (0)
DSA	4	4	4	0	0	3	3	2	2
STA	4	4	4	1	1	3	3	1	2
SBA	0 (2)	0 (2)	0 (2)	0 (0)	0 (0)	0 (1)	0 (1)	0 (0)	0 (0)
DBA	0	0	0	0	0	0	0	0	0
WFA	0	0	0	0	0	0	3	0	0
AHA	0 (2)	0 (2)	0 (1)	0 (1)	0 (0)	0 (3)	2 (3)	0 (3)	2 (3)
MPA	2	0	0	1	1	2	2	3	3
Jac	3 (2)	3 (2)	3 (2)	3 (2)	3 (2)	4 (3)	4 (3)	4 (3)	4 (3)
BSA-1B4	0	0	0	0	0	0	0	0	0
ECA	0 (3)	0 (2)	0 (2)	0 (0)	0 (0)	0 (3)	0 (3)	0 (3)	0 (3)
UEA-1	0	0	0	0	0	0	0	0	0

Key: 0 indicates no binding, 1indicates weak, 2=moderate, 3=moderately strong and 4=intense binding; results in brackets indicate intensity of binding after neuraminidase pretreatment. Abbreviations: RC is rod cytoplasm, CC is cone cytoplasm, RM is rod cell membrane, CM is cone cell membrane, OS is outer segments.

**Figure 2:**
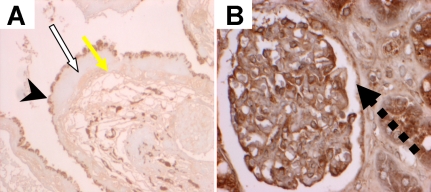
Controls. Panel **A** is negative control. Drusen (indicated by arrow in AMD) shows no binding when the lectin was replaced by 0.05 M TBS with mM calcium chloride. Arrowhead indicates RPE. Panel **B** is positive control. Normal kidney (glomerulus indicated by dashed arrow) stains with CON A.

Pretreatment with neuraminidase did not affect SNA-1 binding to drusen, RPE, and Bruch’s membrane but eliminated binding to photoreceptors; MAA binding to drusen and RPE was reduced after neuraminidase pretreatment, but photoreceptor binding persisted. Neuraminidase pretreatment eliminated LFA and reduced Jac binding while SBA, AHA (Figures 1G,H), and ECA bound or showed increased binding after neuraminidase pre-treatment to drusen, RPE, Bruch’s membrane and photoreceptors. No binding was detected when the lectin was replaced by 0.05M TBS with added calcium with 1 mM calcium chloride (Figure 2A; negative control). Normal kidney glomerulus and tubular epithelial cells stained with CON A (Figure 2B; positive control).

## Discussion

This study demonstrates there are similarities among the lectin binding properties of drusen, RPE, Bruch’s membrane, and photoreceptors, both rods and cones. Lectins with an affinity for high mannose and intermediate structures (CON A) and non-bisected (PSA) and bisected (ePHA) bi and tri-antennary complex N-glycan bound drusen, RPE, Bruch’s membrane and the cell membrane and outer segments of both the rods and cones. Binding of more heavily branched non-bisected complex N-glycan was absent (lPHA). Sialic acid in α2,6 linkage (SNA-1) was detected in drusen, RPE, Bruch’s membrane, and outer segments of rods and cones while sialic acid in α2,3 linkage (MAA) was found in the cores of drusen, RPE, and both the cell membranes and the outer segments of the rods and cones. Other sialic acids, bound by LFA, had a more restricted distribution, being limited to drusen and the outer segments of rods and cones; weak staining was, however, found elsewhere. GlcNAc oligomers (DSA and STA) were present in drusen, RPE, Bruch’s membrane and photoreceptors. Subterminal αGalNAc shown by the binding of SBA after neuraminidase was localized to drusen, RPE, Bruch’s membrane, and outer segments but not to the cell membranes of the photoreceptors. GalNAc in more complex linkages (DBA) and fucose (UEA-1) were not detected in any of the above structures, studied though β−linked galactose bound by MPA, and subterminal to sialic acid (AHA and ECA after neuraminidase) was present in all. Occasional terminal galactosyl residues were also found—for example in the cone outer segments and cell membranes (AHA); this specific feature was abolished with neuraminidase treatment and has also previously been described by Kivela [14]. Cone outer segments were also the only structures that expressed αGalNAc bound by WFA. This study thus shows that, overall, there are striking similarities between the lectin binding of drusen, RPE cells, and the photoreceptor outer segments, though cone outer segments were somewhat distinct in some features of their O-linked glycosylation.

These results are similar to those of Bishop et al. [5] and also correlate with those of Fong et al. [6]. In our study, Jacalin bound drusen and photoreceptors with moderate to strong intensity; removal of sialic acid resulted in reduction in intensity of binding, consistent with Uehara’s findings [7]. We believe that the associated comorbidity does not affect the findings. We have previously shown that drusen associated with various pathologies including AMD, glaucoma, and inflammation, have a similar glycoconjugate distribution. The lectin binding pattern of drusen detected in our study was similar to that observed by Mullins and colleagues [15] in a study of 40 donor eyes ranging from 45 to 96 years. No other ocular or systemic pathology was described in their study.

Visual pigment rhodopsin is embedded in the lipid membranous discs of rod outer segments while cone outer segments contain three different opsins with different peak absorption spectra. Cone pigments are unstable and in low concentration and therefore difficult to isolate. Chicken iodopsin has been isolated and has been shown to contain N-acetyl galactosamine [16]. However there are species variations in the glycosylation patterns of photoreceptors. Human rhodopsin is known to be a glycoprotein containing mannose, N-acetyl glucosamine, and galactose [17]. Rhodopsin is a constituent of over 70% of the rod outer segment protein [18] and thus may account for much of the lectin binding described here; it is broken down by enzymes normally present in the RPE [19]. However, age-related decreases in α-mannosidase, N-acetyl-β-glucosaminidase, and β-galactosidase activity in the RPE [20] would result in incomplete degradation of the outer segments of discs resulting in accumulation of the material as drusen.

With aging, the outer segments of photoreceptors become convoluted and lipofuscin accumulates in the inner segment. RPE cells reduce in number, become pleomorphic, undergo atrophy, hypertrophy, hyperplasia, and cell migration. Bruch’s membrane becomes thickened, basophilic, hyaline and the lipid content increases. The RPE is unable to cope with the phagocytosis of outer segments as well as its own high metabolic needs. Debris accumulates between the RPE and Bruch’s membrane as drusen, which further interfere with the metabolic process leading ultimately to cell death. In keeping with Farkas’ observation [4] that drusen are derived from degenerating RPE cells containing abundant photoreceptor remnants, we propose that the similarities between the lectin-binding properties of drusen, RPE, Bruch’s membrane, and photoreceptors demonstrated here support the hypothesis that the pathogenesis of drusen is a combined mechanism, involving photoreceptors, Bruch’s membrane, and RPE.
